# Supplementary material for: Giant DNA viruses encode a hallmark translation initiation complex of eukaryotic life
Source: bioRxiv. 2025 Sep 30:2025.09.30.678621. Preprint. [Version 1] doi: 10.1101/2025.09.30.678621 (PMC12621921; doi:10.1101/2025.09.30.678621)
Supplement: 1 [file NIHPP2025.09.30.678621V1-supplement-1.pdf]

bioRxiv preprint doi: https://doi.org/10.1101/2025.09.30.678621; this version posted September 30, 2025. The copyright holder for this preprint (which was not certified by peer review) is the author/funder, who has granted bioRxiv a license to display the preprint in perpetuity. It is made available under aCC-BY 4.0 International license.

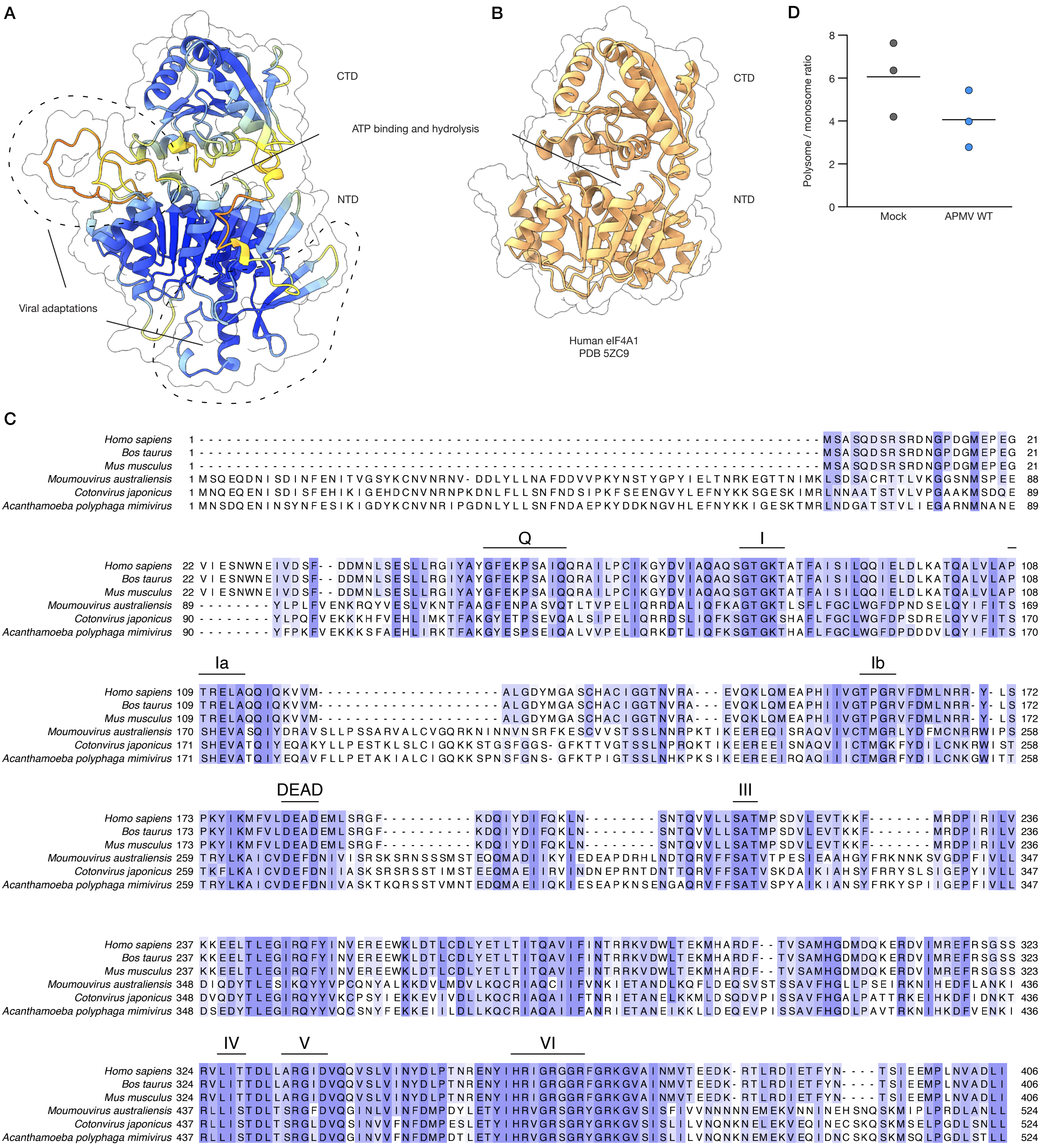

Figure S1. vIF4A is DEAD-box helicase with virus-specific features. A. AlphaFold2 model of APMV vIF4A colored by pLDDT. Virus-specific extensions are highlighted. **B.** Crystal structure of human eIF4A1(PDB 5ZC9) illustrating the conserved nature of the two-domain helicase fold. **C.** Multiple sequence alignment of vIF4A and eIF4A colored by % identity. The functional motifs of DEAD-box helicases are highlighted. **D.** Polysome / monosome ratios for mock-infected and APMV WT-infected cells. The line represents the mean of three biological replicates. Related to Figure 1.

bioRxiv preprint doi: https://doi.org/10.1101/2025.09.30.678621; this version posted September 30, 2025. The copyright holder for this preprint (which was not certified by peer review) is the author/funder, who has granted bioRxiv a license to display the preprint in perpetuity. It is made available under aCC-BY 4.0 International license.

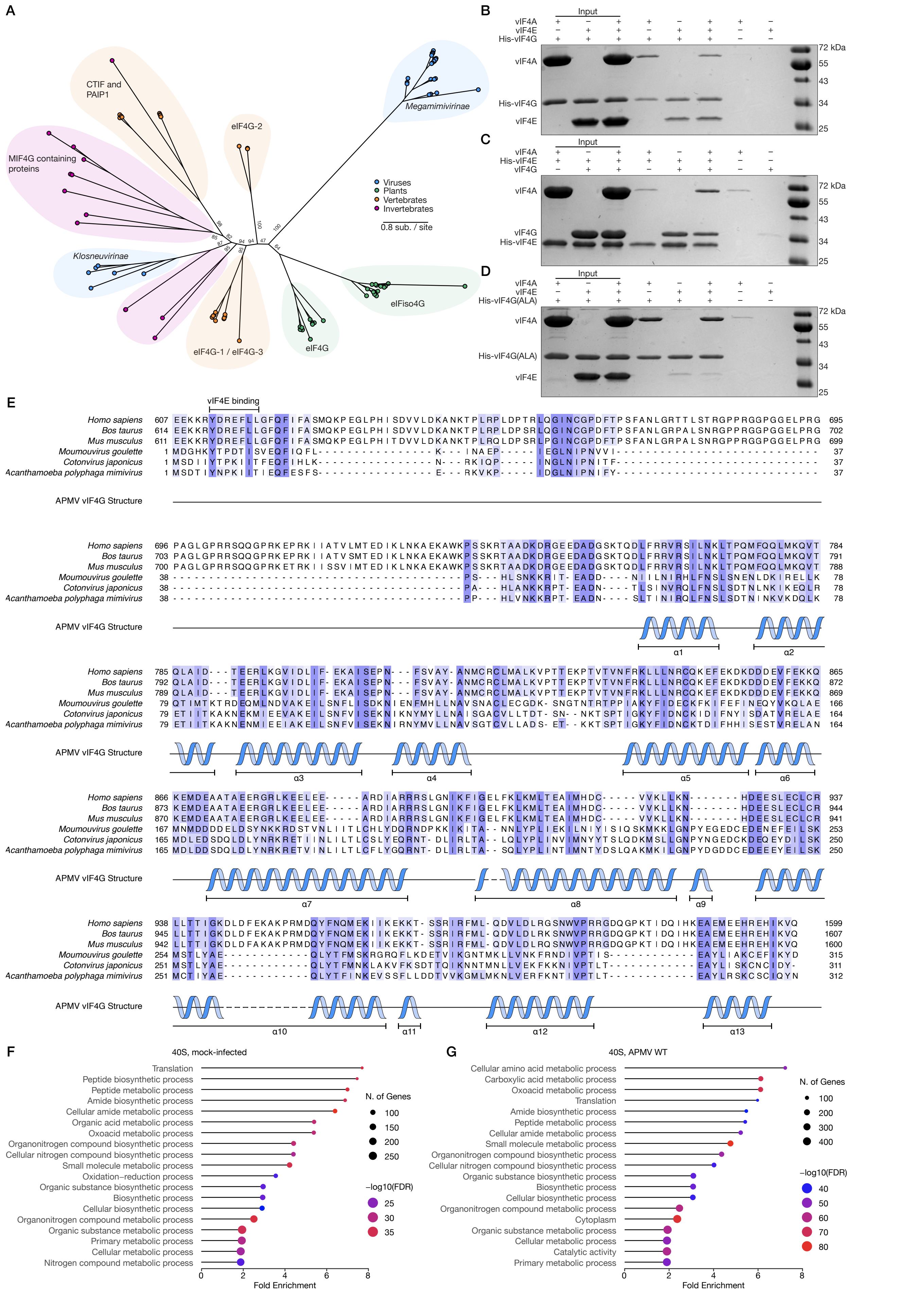

bioRxiv preprint doi: https://doi.org/10.1101/2025.09.30.678621; this version posted September 30, 2025. The copyright holder for this preprint (which was not certified by peer review) is the author/funder, who has granted bioRxiv a license to display the preprint in perpetuity. It is made available under aCC-BY 4.0 International license.

Figure S2. Formation of a viral IF4F complex by a phylogenetically distinct vIF4G. A. Phylogenetic tree of eukaryotic and viral MIF4G-like proteins illustrating the divergent nature of APMV vIF4G. Branch supports are provided for the basal branches. **B.** Expanded version of Fig. 1F that includes input lanes and no-bait controls. **C.** vIF4F complex formation mediated by His-vIF4E as visualized by Coomassie-stained SDS-PAGE gel. **D.** Expanded cropped version of Fig. 1G that includes input lanes and no-bait controls. **E.** Multiple sequence alignment of the MIF4G domains from eIF4G1 and vIF4G homologs colored by % identity. The main structural features of APMV vIF4G are aligned with the sequence. **F.** GO enrichment analysis (biological process) of host proteins associated with 40S fractions in mock-infected cells. **G.** GO enrichment analysis (biological process) of host proteins associated with 40S fractions in cells infected by APMV WT. Related to Figure 1.

bioRxiv preprint doi: https://doi.org/10.1101/2025.09.30.678621; this version posted September 30, 2025. The copyright holder for this preprint (which was not certified by peer review) is the author/funder, who has granted bioRxiv a license to display the preprint in perpetuity. It is made available under aCC-BY 4.0 International license.

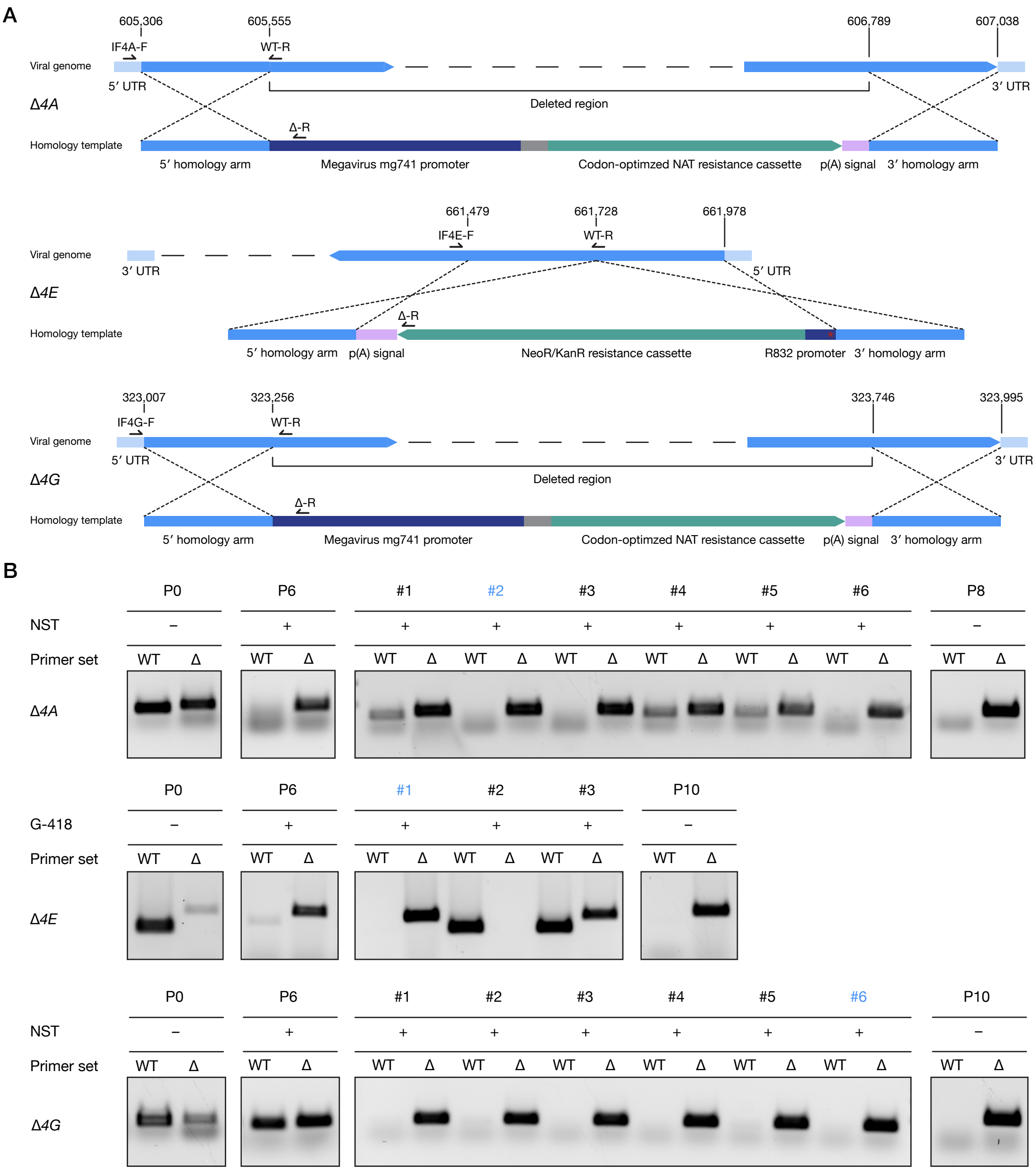

Figure S3. Knock-out of APMV IF4F subunits. **A.** Design of constructs used to generate *Δ4A, Δ4E*, and *Δ4G* APMV. The red star in the R832 promoter indicates a premature stop codon. **B.** Diagnostic PCR of *Δ4A, Δ4E*, and *Δ4G* APMV. The clonal populations selected after limiting dilution are indicated in blue. Related to Figure 2.

bioRxiv preprint doi: https://doi.org/10.1101/2025.09.30.678621; this version posted September 30, 2025. The copyright holder for this preprint (which was not certified by peer review) is the author/funder, who has granted bioRxiv a license to display the preprint in perpetuity. It is made available under aCC-BY 4.0 International license.

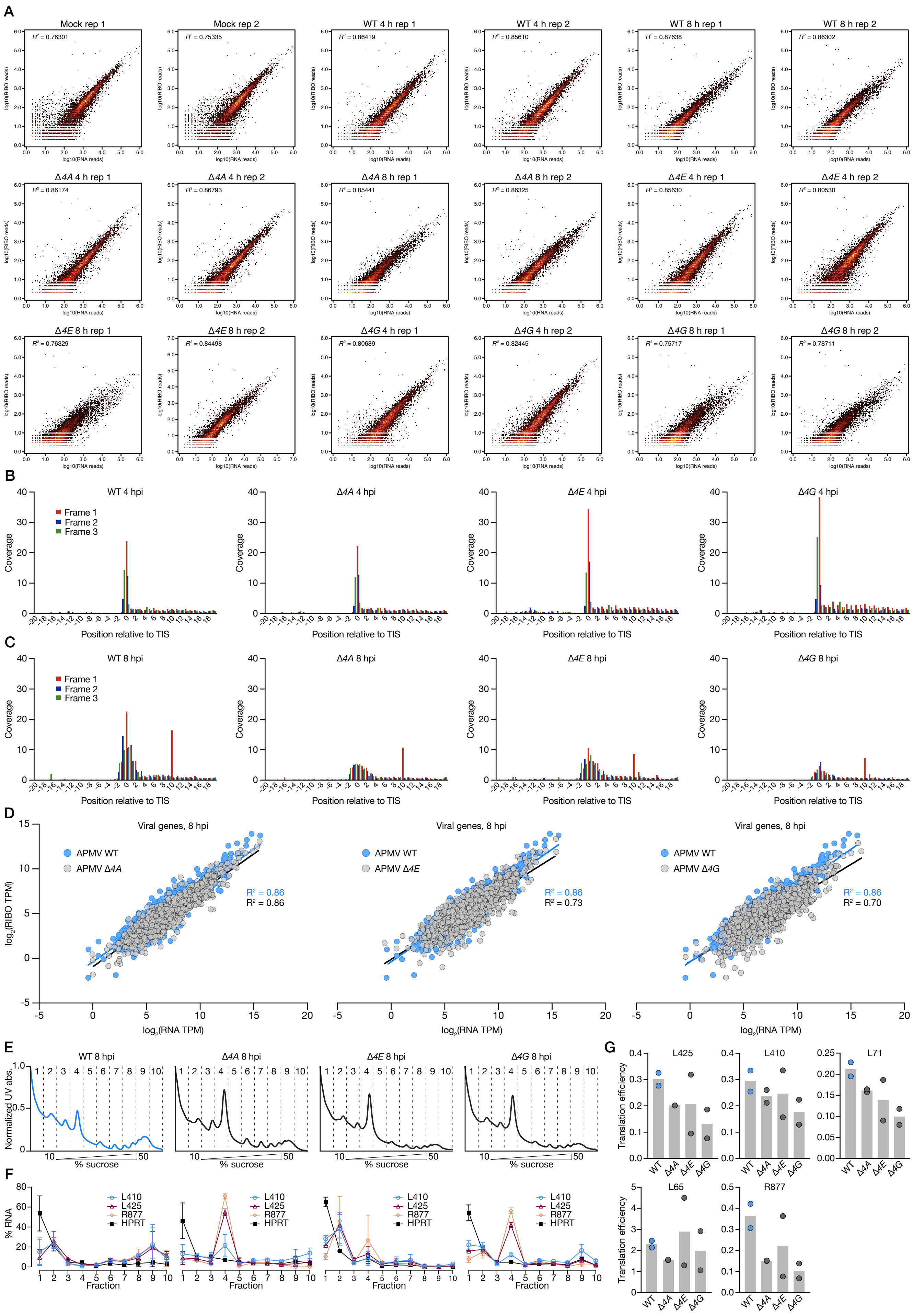

bioRxiv preprint doi: https://doi.org/10.1101/2025.09.30.678621; this version posted September 30, 2025. The copyright holder for this preprint (which was not certified by peer review) is the author/funder, who has granted bioRxiv a license to display the preprint in perpetuity. It is made available under aCC-BY 4.0 International license.

Figure S4. Ribosome profiling quality control and metagene analysis. A. Congruence plots of log_{10}-transformed RNA-seq and RIBO-seq transcript abundances across samples. Individual replicates are plotted separately. **B.** Metagene analysis, centered on the start codon, of viral genes from WT *Δ4A, Δ4E*, and *Δ4G* APMV at 4 hpi. **C.** Metagene analysis, centered on the start codon, of viral genes from WT *Δ4A, Δ4E*, and *Δ4G* APMV at 8 hpi. **D.** Normalized and log_2-transformed RNA and RPF abundances of all viral transcripts detected at 8 hpi during APMV *Δ4A, Δ4E*, and *Δ4G* infection. Distributions of the mutants are overlaid onto that of APMV WT for visualization purposes. Each dot represents the mean of two biological replicates and solid lines represent a linear fit of the data with the associated R^2 values. **E.** Representative examples of polysome profiles from cells infected with APMV WT, *Δ4A, Δ4E*, or *Δ4G* collected at 8 hpi. Sucrose gradient fractions are marked by dashed lines. **F.** % of target mRNAs associated with each sucrose gradient fraction from cells infected with APMV WT, *Δ4A, Δ4E*, or *Δ4G* at 8 hpi. Symbols and lines represent the mean +/- range from two biological replicates. **G.** Translation efficiency (RIBO-seq : RNA-seq ratio) for each of the selected structural proteins in cells infected with APMV WT, *Δ4A, Δ4E*, or *Δ4G* at 8 hpi. Each bar represents the mean of two biological replicates. Related to Figure 3.

bioRxiv preprint doi: https://doi.org/10.1101/2025.09.30.678621; this version posted September 30, 2025. The copyright holder for this preprint (which was not certified by peer review) is the author/funder, who has granted bioRxiv a license to display the preprint in perpetuity. It is made available under aCC-BY 4.0 International license.

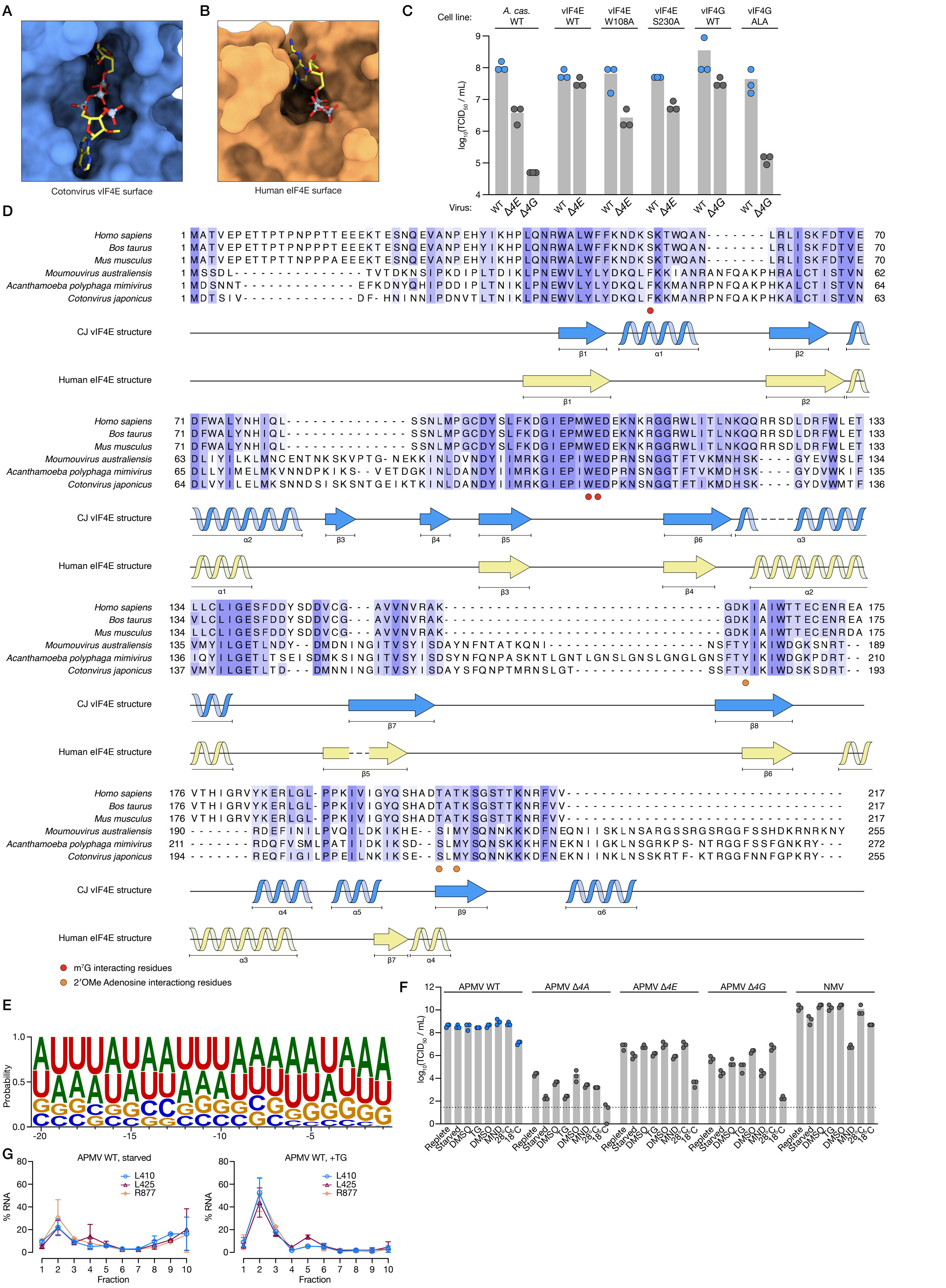

bioRxiv preprint doi: https://doi.org/10.1101/2025.09.30.678621; this version posted September 30, 2025. The copyright holder for this preprint (which was not certified by peer review) is the author/funder, who has granted bioRxiv a license to display the preprint in perpetuity. It is made available under aCC-BY 4.0 International license.

Figure S5. Structural and sequence comparison of vIF4E and human eIF4E. **A.** Surface representation of vIF4E from CJ in complex with m^7Gppp(2′OMeA)pU demonstrating the close coordination of the ligand. **B.** Surface representation of human eIF4E in complex with m^7Gppp (PDB 5T46). **C.** Viral replication in *A. castellanii* cell lines expressing mutant or WT forms of vIF4E and vIF4G. Each bar represents the mean of three biological replicates. All residue numbers correspond to APMV vIF4E. **D.** Multiple sequence alignment of the vIF4E and eIF4E homologs colored by % identity. The main structural features of CJ vIF4E and human eIF4E are aligned with the sequence. **E.** Weblogo of the 20 nt upstream of the AUG in NMV intergenic regions. **F.** Raw titers of APMV WT, *Δ4A, Δ4E, Δ4G*, or NMV replication under stress conditions. The limit of detection is indicated by a dotted line. **G.** % of target mRNAs associated with each sucrose gradient fraction from cells infected with APMV WT under stravation or TG treatment 8 hpi. Related to Figure 4 and 5.
